# Sorting receptor SORLA: cellular mechanisms and implications for disease

**DOI:** 10.1007/s00018-016-2410-z

**Published:** 2016-11-10

**Authors:** Vanessa Schmidt, Aygul Subkhangulova, Thomas E. Willnow

**Affiliations:** 1 Max-Delbrueck-Center for Molecular Medicine, Robert-Roessle-Str. 10, 13125 Berlin, Germany; 2 Berlin Institute of Health, Berlin, Germany

**Keywords:** VPS10P domain receptors, Protein sorting, Retromer, GGA, Alzheimer’s disease, Obesity

## Abstract

Sorting-related receptor with A-type repeats (SORLA) is an intracellular sorting receptor that directs cargo proteins, such as kinases, phosphatases, and signaling receptors, to their correct location within the cell. The activity of SORLA assures proper function of cells and tissues, and receptor dysfunction is the underlying cause of common human malignancies, including Alzheimer’s disease, atherosclerosis, and obesity. Here, we discuss the molecular mechanisms that govern sorting of SORLA and its cargo in multiple cell types, and why genetic defects in this receptor results in devastating diseases.

## Introduction

Sorting of proteins to their destined location in subcellular compartments is essential for proper cell function, and faulty protein sorting will result in cellular dysfunction and disease. Protein sorting is essential for all cell types, but particularly challenging in neurons in which cell compartments of axons and dendrites may be as far away as 1 m from the soma of motor neurons. Within cells, the Golgi is the central hub that sorts the bulk of proteins. Protein sorting proceeds in the *trans*-most cisterna of this organelle called the *trans*-Golgi network (TGN) that consists of an elaborate web of branching tubular membrane domains. From the TGN, proteins may be targeted to the apical or basolateral plasma membranes, to the endosomal/lysosomal system, or to specialized secretory granules for activity-dependent release. Directed protein trafficking is mediated by sorting receptors, transmembrane proteins that interact with cytosolic adaptors at the Golgi membranes to guide their protein cargo to and from the TGN (reviewed in [1]).

In recent years, one group of sorting receptors received particular attention because of their causal involvement in human diseases, such as Alzheimer’s and Huntington’s disease, psychiatric disorders, but also atherosclerosis, dyslipidemia, and diabetes. These sorting receptors are called VPS10P domain receptors. Initially characterized in neurons in the brain, VPS10P domain receptors now emerge as key regulators of intracellular protein sorting not only in the nervous system but also in many other tissues as well [2]. Here, we focus on the sorting-related receptor with A-type repeats (SORLA), a VPS10P domain receptor that is paradigmatic for the mode of action of this class of sorting receptors. We describe the molecular interactions of SORLA with adaptor complexes that control traffic between TGN, plasma membrane, and endosomes. We discuss the functional significance of this trafficking pathway for proper routing of enzymes, growth factors, and signaling receptors, and why SORLA dysfunction may result in devastating pathologies, including neurodegeneration, impaired renal ion homeostasis, and obesity.

## Cell biology of SORLA sorting

**Fig. 1:**
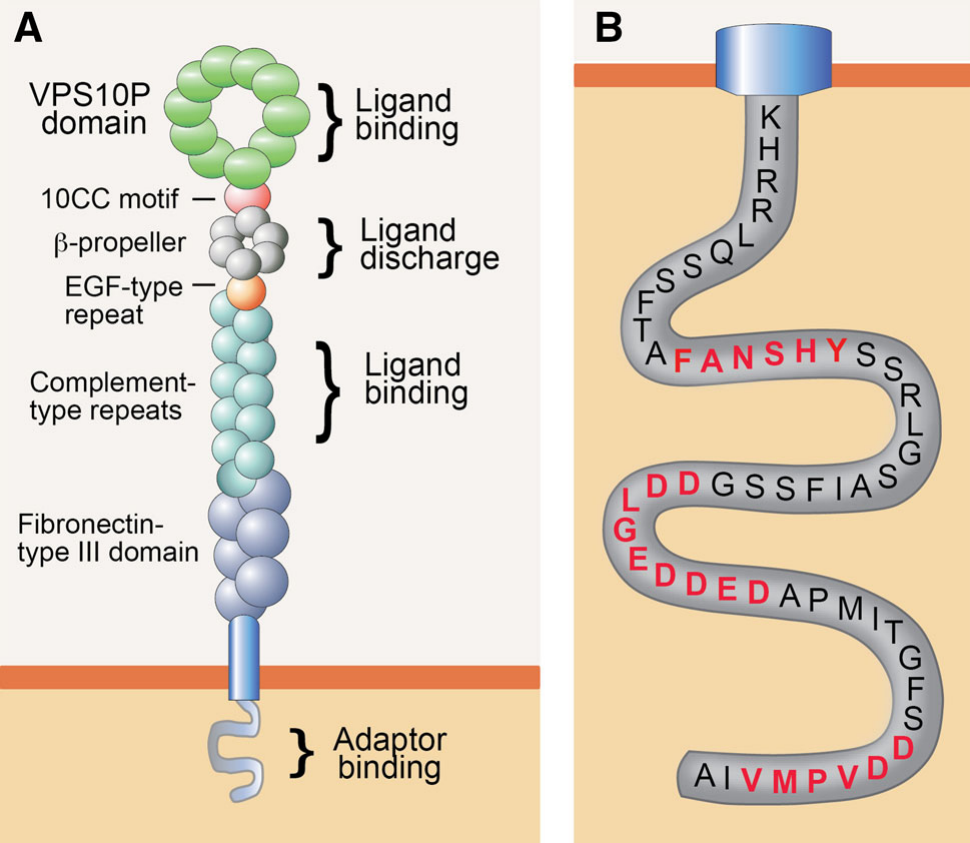
Structural organization of SORLA. **a** Organization of the SORLA polypeptide is shown, indicating the main structural elements and their documented functions. The VPS10P domain and the cluster of complement-type repeats serve as major ligand-binding sites in the luminal receptor domain. The β-propeller interacts with the molecular chaperone MESD to facilitate folding of the receptor polypeptide, and it may be involved in pH-dependent release of bound ligands in acidic endosomal compartments. **b** Amino-acid sequence of the cytoplasmic receptor tail highlighting three main binding motifs for cytosolic adaptors, termed FANSHY, the acidic motif (DDLGEDDED), and the GGA-binding site (DDVPMV). GGA, Golgi-localizing, γ-adaptin ear homology domain, ARF-interacting protein; MESD, mesodermal development deletion interval; VPS10P, vacuolar protein sorting 10 proteins. **a** adapted from [43]

SORLA (also known as LR11 or SORL1) was initially uncovered in the search for receptors that share structural similarity to the low-density lipoprotein (LDL) receptor, the main endocytic receptor for uptake of lipid-loaded lipoproteins into vertebrate cells. These studies identified a 250-kDa type 1 transmembrane protein in brain [3] and liver [4] that contained complement-type repeats and a β-propeller, structural elements in the LDL receptor required for binding and for pH-dependent release of ligands, respectively (Fig. 1a). The ability of SORLA to internalize lipoproteins seemingly supported the notion of a novel species of lipoprotein receptor [5, 6]. However, this assumption was questioned by the presence of additional structural elements in SORLA not found in the LDL receptor, namely a VPS10P domain and six fibronectin-type III domains (Fig. 1a). The VPS10P domain was noteworthy as it was identified earlier in an intracellular sorting protein in yeast called the vacuolar protein sorting 10 proteins (VPS10P) [7]. VPS10P directs newly synthesized peptidases from the TGN to the vacuole (the yeast lysosome) where they act in proteolytic breakdown of internalized proteins. A similar function for SORLA in intracellular protein sorting in mammalian cell types was supported by the fact that the bulk of the receptor molecules was present in the Golgi rather than at the cell surface, a finding that argued against a role as endocytic receptor [6, 8].

By now, an extensive body of work has substantiated the relevance of SORLA as an intracellular sorting receptor that shuttles between TGN, cell surface, and endosomes in neurons and multiple other cell types. SORLA is synthesized as a pro-receptor containing a 53 amino-acid pro-peptide at the ultimate amino terminus. This pro-peptide is believed to block the binding site for ligands in the VPS10P domain, a major site for interaction with peptide ligands [9, 10]. Removal of the pro-peptide by convertases in the TGN activates the ligand-binding capability of the receptor [6]. This activation step may be required to prevent premature binding of ligands to nascent receptor molecules in the biosynthetic pathway of the cell. Apart from the VPS10P domain, the cluster of complement-type repeats in SORLA constitutes another site for ligand recognition [11, 12]. Binding of ligands to the VPS10P domain or the complement-type repeats is lost at low pH (<5.5) [13], suggesting ligand interaction to partake in the secretory pathway and at the cell surface, but to be disrupted in the acidic milieu of late endosomes. The significance of additional structural elements in the extracellular domain for receptor functions is less clear. Based on analogy to other proteins, the fibronectin-type III domain may be involved in protein–protein interactions [14], while the β-propeller may facilitate pH-dependent release of ligands in endocytic compartments [15].

**Fig. 2:**
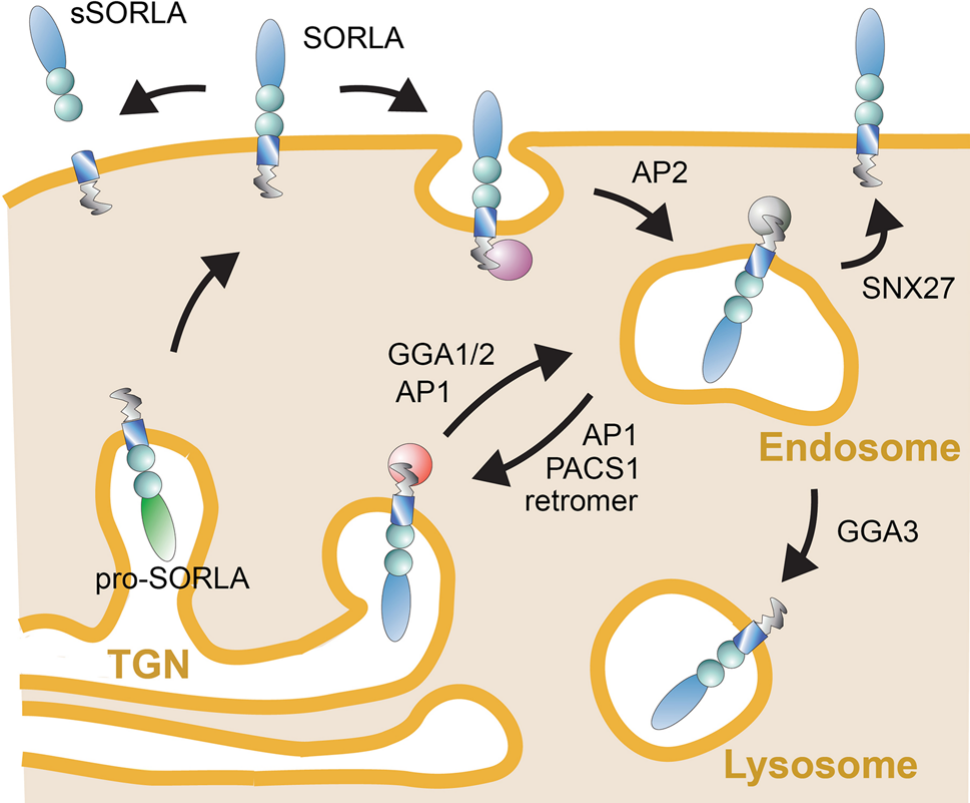
Intracellular trafficking path for SORLA. Nascent SORLA is an inactive pro-receptor (pro-SORLA) that is activated by proteolytic removal of an amino-terminal pro-peptide in the TGN, resulting in transfer of the active receptor (SORLA) through the constitutive secretory pathway to the cell surface. Some receptor molecules at the cell surface are subjected to ectodomain shedding, resulting in release of the extracellular receptor domain. Ectodomain shedding disrupts the ability of SORLA to act as a sorting receptor, but may serve to produce a soluble receptor fragment termed soluble (s)SORLA that acts as a signaling molecule. Still, most SORLA molecules at the cell surface remain intact and undergo clathrin-dependent endocytosis facilitated by the clathrin adaptor protein (AP) 2. The bulk of internalized receptors move from endosomes back to the TGN to continuously shuttle between TGN and endosomal compartments thereafter. Adaptors GGA1 and GGA2 guide anterograde movement of SORLA from the TGN to endosomes, whereas PACS1 and the retromer complex facilitate retrograde sorting from endosomes back to the Golgi. AP1 may be involved in bi-directional sorting. As alternative routes, SORLA may sort from endosomes to the cell surface (aided by adaptor SNX27) or to lysosomes (aided by GGA3). Figure adapted from [43]. AP, adaptor protein; GGA, Golgi-localizing, γ-adaptin ear homology domain, ARF-interacting protein; PACS1, phosphofurin acidic cluster sorting protein 1; SNX27, sorting nexin family member 27

As well as by the ability to recognize distinct ligands through its luminal domain, the function of SORLA in protein sorting is determined by information encoded in its short cytoplasmic tail. This tail domain encodes recognition motifs for cytosolic adaptors that direct the complex trafficking path of SORLA between cell surface and various intracellular compartments (Fig. 1b). Typically, newly synthesized SORLA molecules follow the constitute secretory pathway from the endoplasmic reticulum through the Golgi to the cell surface, a default route for transmembrane proteins that does not require distinct sorting motifs (Fig. 2). At the cell surface, some SORLA molecules are subject to proteolytic shedding releasing the soluble ectodomain of the receptor, termed soluble (s) SORLA [16, 17]. However, most SORLA molecules at the cell surface remain intact and undergo clathrin-dependent endocytosis guided by the clathrin adaptor protein 2 (AP2) that interacts with an acidic motif D^2190^DLGEDDED in the receptor tail [8]. Internalized receptors move to the early endosomes from where most receptors will sort to the TGN to continuously shuttle between TGN and endosomes thereafter. Retrograde movement of SORLA from endosomes to the TGN is guided by phosphofurin acidic cluster sorting protein 1 (PACS1) that also binds to the acidic cluster [18], and by the multimeric adaptor complex retromer that binds to the F^2172^ANSHY tail motif [19, 20]. Anterograde sorting of SORLA from the TGN to endosomes is mediated by the monomeric clathrin adaptors GGA1 and GGA2 (Golgi-localizing, γ-adaptin ear homology domain, and ARF-interacting proteins) that interact with the D^2207^DVPMVIA element in the SORLA tail [18, 21, 22]. Finally, binding of the adaptor protein AP1 to the acidic tail motif may aid in anterograde as well as retrograde sorting of SORLA [8]. The shuttling of protein cargo between TGN and endosomes likely constitutes the major trafficking route taken by SORLA in neurons. However, some studies also report the ability of SORLA to move ligands from endosomes to the cell surface guided by the sorting nexin family member (SNX) 27 [23], or from endosomes to lysosomes, potentially sorted by GGA3 [24, 25]. The complex trafficking path for SORLA has mainly been elucidated in established cell lines. However, recent studies in mouse models expressing mutant SORLA variants lacking individual adaptor binding sites have substantiated this model in the brain by documenting impaired anterograde sorting in receptor mutants lacking the GGA-binding site [25] and impaired retrograde sorting in mutants unable to interact with PACS1 [26] or retromer [25].

## SORLA controls amyloidogenic processes in the brain

Given the predominant expression of SORLA in the brain and its complex trafficking path in neurons, major efforts have been focused on identifying the protein cargo sorted by this receptor and its relevance for brain (patho)physiology. These studies have highlighted an important role for SORLA in control of amyloidogenic processes in the brain and as a major risk factor for Alzheimer’s disease (AD).

Central to the pathology of AD is the amyloid precursor protein (APP), a type-1 transmembrane protein expressed in many cell types, including neurons. In a naturally occurring process, APP is broken down into various proteolytic fragments, including the amyloid-β peptides (Aβ), peptides of 37–43 amino-acid length that encompass part of the transmembrane, and extracellular domains of APP. Amyloid-β peptides, notably Aβ42, are considered main culprits in neurodegenerative processes as they exhibit a tendency to aggregate to neurotoxic oligomers and senile plaques, pathological features causative of neuronal dysfunction and cell loss in AD patients (reviewed in [27]). Amyloidogenic processing requires endocytosis of APP molecules from the cell surface and delivery to endosomes whereby proteolytic breakdown to Aβ occurs [28–30]. As it turns out, SORLA acts as a sorting receptor for APP that shuttles internalized precursor molecules from endosomes back to the TGN to decrease production of Aβ [18, 31, 32]. Binding of APP proceeds through the cluster of complement-type repeats in SORLA that forms a 1:1 stoichiometric complex with the luminal domain of APP [11, 12, 33]. Overexpression of SORLA in cells reduces Aβ formation [18, 31, 32, 34], while loss of expression accelerates Aβ production and senile plaque deposition [35, 36], documenting a protective function for SORLA in AD progression. The interaction of SORLA and APP is blocked by signaling through β-adrenergic receptors via a yet unknown mechanism, resulting in impaired Golgi retrieval and in increased endosomal accumulation of APP [37]. In line with the presumed sorting path of the receptor, prevention of APP processing depends on the ability of SORLA to move retrogradely from endosomes to the TGN and is lost in receptor mutants that cannot interact with retromer [25] or PACS1 [26]. As well as by sorting APP, SORLA has also been shown to reduce the amyloidogenic burden by sorting of newly produced Aβ peptides to lysosomes for catabolism [13]. This activity depends on the interaction of Aβ with a recognition site in the VPS10P domain of SORLA [10] and on the ability of the receptor to interact with GGAs to move in an anterograde fashion from the TGN to endocytic compartments [25].

Taken together, the ability of SORLA to sort APP and Aβ likely represents major mechanisms, whereby this receptor reduces the amyloidogenic burden and delays progression of neurodegeneration. This hypothesis received strong support from genetic studies in AD patients that identified gene variants in *SORL1*, the gene encoding SORLA, as being associated with the risk of the sporadic form of AD [34, 38, 39]. Some of these sequence variants have been shown to impair efficiency of *SORL1* transcription [34, 40, 41] or translation [42], in line with low levels of SORLA being disease promoting in patients and mouse models (reviewed in [43]). Furthermore, a missense mutation in *SORL1*, that disrupts its ability to bind Aβ, has been identified in a family with the autosomal dominant form of AD [44].

## SORLA in neurotrophin signaling

Protein cargo sorted by SORLA in neurons is not restricted to APP and its processing products, but also encompasses a number of neurotrophin receptors, cell surface proteins that transmit trophic signals to support growth and survival of neurons. Specifically, SORLA acts as a sorting factor for the tropomyosin-related kinase receptor (TrkB), the receptor for brain-derived neurotrophic factor (BDNF). SORLA facilitates trafficking of TrkB between synaptic membranes and the cell soma, a step critical for BDNF signal transduction into cells [45]. Loss of SORLA results in impaired neuritic transport of TrkB and in a blunted response to BDNF [45]. Intriguingly, SORLA is also a downstream target of BDNF with receptor gene transcription being induced almost 10-fold by BDNF signaling in neurons [41, 46]. These data suggest a positive feedback loop, whereby BDNF enhances trophic signaling through induction of *SORL1*, the gene encoding the sorting receptor for TrkB.

Another trophic pathway modulated by SORLA acts through glial cell-line-derived neurotrophic factor (GDNF) that promotes survival of distinct populations of central and peripheral neurons, such as midbrain dopaminergic neurons and spinal motor neurons. SORLA interacts with GDNF to increase its regulated secretion from cells [47]. In addition, SORLA interacts with GFRα1, the co-receptor for GDNF [48]. SORLA facilitates internalization of GFRα1/GDNF complexes from the plasma membrane, resulting in lysosomal catabolism of GDNF but cell surface recycling of GFRα1. This sorting route provides an efficient pathway for clearance of GDNF from the extracellular space and counteracts consequences of excessive GDNF signaling, such as hyperactivity and reduced anxiety (as seen in mice lacking SORLA) [48]. Finally, SORLA also impacts signaling through a heterodimeric neurotrophic cytokine called cardiotrophin-like cytokine:cytokine-like factor 1 (CLC:CLF-1) [49]. Specifically, SORLA interacts with the CLF-1 moiety to facilitate internalization of the cytokine in complex with the ciliary neurotrophic factor receptor (CNTFR) α. SORLA-dependent endocytosis is required for neurotrophic signaling through CLC:CLF-1, but it also downregulates signal reception by directing ligand and receptor to lysosomal degradation [49].

The cell biology of SORLA-dependent sorting of neurotrophins and their receptors, and the implication of cytosolic adaptors in this process, still awaits further investigation. However, the ability of this receptor to impact pathways both for trophic support but also of amyloidogenic insult to neurons makes this sorting pathway an important target in control of neurodegenerative processes in patients.

## SORLA in renal ion homeostasis

While major attention has been focused on the relevance of SORLA for protein sorting in neurons, other studies have uncovered important roles for this protein in non-neuronal cell types, as well. Thus, SORLA is abundantly expressed in the thick ascending limb (TAL) of Henle’s loop, a distal segment of the renal nephron responsible for water and ion homeostasis [50, 51]. Lack of SORLA expression in epithelial cells of the TAL results in failure to properly reabsorb sodium and chloride, a defect attributed to the inability of these cells to activate the major sodium transporter in the distal nephron Na–K-Cl cotransporter 2 (NKCC2) [51]. As it turns out, SORLA controls the phospho-regulation of NKCC2 by interacting with both the Ste-20-related proline-alanine-rich kinase (SPAK) [51] and the calcineurin phosphatase [52] that carry out phosphorylation and dephosphorylation of NKCC2, respectively. These findings suggest SORLA-mediated sorting of kinases and phosphatases as a regulatory process in modulation of renal ion balance.

## SORLA in vascular cell migration and atherosclerosis

Atherosclerosis, or thickening of the artery wall, is a major risk factor for cardiovascular morbidity and mortality, including myocardial infarction and stroke. Atherosclerosis is caused by excessive accumulation of lipids in macrophages in the vessel wall (foam cells) and by the proliferation of intimal smooth muscle cells. Jointly, these processes contribute to the formation of fibrous plaques that may obstruct the vessel lumen. Interestingly, *Sorl1* has been mapped as a pro-atherogenic locus in mice [53]. The relevance of SORLA for atherosclerotic processes was further supported by correlating circulating levels of the shedded ectodomain sSORLA (indicative of full-length receptor levels in tissues) with intima-media thickness in subjects with coronary artery disease [54] or acute coronary syndrome [55]. Currently, there are two main hypotheses how SORLA impacts atherosclerotic plaque formation. One model suggests a role for SORLA in control of plasma triacylglyceride levels through regulation of lipolysis. Triacylglyceride-rich lipoproteins are highly pro-atherogenic particles. Their turnover is determined by hydrolysis of triacylglycerides to free fatty acids through lipoprotein lipase (LPL) in the circulation. SORLA traffics newly synthesized LPL molecules from the TGN to lysosomes, reducing the amount of the enzyme being secreted by cultured cells [56]. In addition, SORLA mediates the endocytosis of apoA-V, an activator of LPL [57, 58]. Modulation of LPL activity through clearance of apoA-V is supported by the loss of SORLA binding in an apoA-V variant found in individuals with severe hypertriglyceridemia [59]. Potentially, either through control of LPL or apoA-V levels, SORLA may inhibit lipolysis and raise the levels of pro-atherogenic lipoprotein particles in the circulation.

An alternative model suggests a more direct role for SORLA in atherosclerotic processes in the vessel wall. It is based on the ability of SORLA to stimulate proliferation and migration of intimal smooth muscle cells (SMC) and monocytes, processes that accelerated intimal thickening and atherosclerotic plaque formation [60–63]. Potentially, the stimulation of SMC migration by SORLA works through modulation of cell surface expression of the urokinase receptor (uPAR) [64, 65]. The uPAR is a glycosylphosphatidyl inositol-anchored receptor for urokinase, a protease that activates plasminogen to plasmin, which, in turn, breaks down the extracellular matrix. Binding of urokinase to uPAR on the surface of cells increases their proteolytic potential and facilitates migration. The ability to regulate surface exposure of uPAR is seen for full-length SORLA but also for sSORLA, suggesting both cell autonomous and non-autonomous modes of action [65, 66].

## SORLA is a risk factor for obesity

Genome-wide association studies not only confirmed the relevance of *SORL1* as a genetic risk factor for sporadic AD [38] but also revealed a surprising association of this locus with metabolic traits (e.g., obesity and waist circumference) in humans and mouse models [67, 68]. In addition, loss of SORLA expression in mice with targeted *Sorl1* disruption is protected from diet-induced obesity, suggesting a so far unknown function for this receptor in metabolic regulation [69, 70]. Recent studies in transgenic mouse models shed light on potential modes of receptor action, albeit proposing distinct roles for SORLA and sSORLA in this context. In a study by Whittle and colleagues, sSORLA was shown to impair thermogenesis in mice by binding to bone morphogenetic protein (BMP) receptors and inhibiting BMP/TGF β signaling in adipocytes [69]. Thermogenesis is the process of heat production from metabolic fuel and a driving force for consumption of body lipid stores by brown adipose tissue. Mice genetically deficient for SORLA are protected from diet-induced obesity because of enhanced thermogenesis in adipose tissue, providing an explanatory model for the association of *SORL1* with obesity in the human population [69].

An alternative model to explain the role of SORLA in energy homeostasis entails intracellular sorting of the insulin receptor (IR) [70]. One of the actions of insulin signaling in adipocytes is the downregulation of lipolysis. This mechanism reduces energy production from breakdown of lipid stores in a state of sufficient energy supply from carbohydrates. Cellular signal transduction proceeds through binding of insulin to the IR on the surface of target cells and subsequent endocytosis of receptor and hormone complexes. Internalization serves two purposes. First, it delivers receptor-ligand complexes to endosomes, a prerequisite for signal transduction. Second, it moves receptor-ligand complexes to lysosomal compartments for catabolism, a mean to downregulate signal reception. In a process reminiscent of APP sorting in neurons, SORLA interacts with internalized IR molecules in endosomes and shuttles them back to the TGN. Retrograde trafficking reduces lysosomal catabolism and increases the fraction of IR molecules recycled back to the cell surface. SORLA-dependent recycling sensitizes adipocytes for insulin signal reception and enhances the impact of insulin on blockade of lipolysis. Consequently, overexpression of SORLA in adipose tissue of mice inhibits lipolysis and promotes the fat mass gain, while loss of the receptor expression increases lipolysis rate and protects animals from obesity and secondary metabolic complications [70].

In obese human subjects, the levels of SORLA in adipose tissue [70] and those of sSORLA in the circulation [69] positively correlate with the body mass index. Although the exact mode of action in adipose tissues as humoral factor or as sorting receptor still awaits further clarification, all current data support the significance of *SORL1* as genetic risk factor of obesity in the human population.

## Outlook: SORLA dysfunction as an explanatory model for comorbidities?

Studies on seemingly unrelated disease entities have converged on SORLA as a sorting receptor for multiple ligands in organs, such diverse as the brain, the kidney, or adipose tissue. These observations raise the intriguing possibility that SORLA (dys)function may explain some of the comorbidities commonly seen in the human population as exemplified for AD and type II diabetes (T2D). T2D, a disease characterized by lack of responsiveness of cells to insulin (insulin resistance), is one of the major risk factors for sporadic AD [71]. As well as in peripheral tissues, such as muscle, liver, and fat, insulin signaling is also widespread in neurons in the brain, where it modulates central control of metabolism but also behavior and memory [72–74]. In AD patients, brain insulin signaling is impaired, partially due to reduced levels of the hormone [75] and abnormal intracellular sequestration of the IR in neurons caused by Aβ [76, 77]. Conversely, alerted levels of insulin signaling in a diabetic state may impact Aβ metabolism by changing rates of production and clearance as suggested by studies in vitro and in rodent models [78, 79]. Currently, the mechanistic link between brain insulin resistance and amyloidogenic processes is a matter of intense debate and the reader is referred to excellent recent reviews in this subject ([80]). A role for SORLA in insulin signaling in neurons has not been documented yet. However, low levels of the receptor are likely to result in decreased neuronal sensitivity to the hormone as shown for adipocytes previously [70]. Although quite speculative at present, low levels of receptor expression in carriers of *SORL1* risk alleles may thus cause both central insulin resistance and increased amyloidogenic burden, and prove to be an explanatory model for the link between neurodegenerative and metabolic diseases that warrant further exploration.
